# Intra-articular injection of methylprednisolone for reducing pain in knee osteoarthritis: A systematic review and meta-analysis: Retraction

**DOI:** 10.1097/MD.0000000000015902

**Published:** 2019-05-24

The article, “Intra-articular injection of methylprednisolone for reducing pain in knee osteoarthritis: A systematic review and meta-analysis”^[1]^, published in Volume 97, Issue 15 of *Medicine* has been retracted by the journal.

After publication of the article, the journal was notified that there were inaccuracies in the review methodology and conclusions. Specifically the inclusion criteria of the article^[1]^ indicates that papers must have investigated methylprednisolone vs placebo. Of the four papers included, two papers^[2,3]^ are not placebo controlled trials. Also, the two correctly identified placebo controlled trials in the meta-analysis^[4,5]^ do not show any benefit for methylprednisolone over placebo at 24 weeks. The heaviest weighting for the reported results in favour of methylprednisolone shown in Figure 3^[1]^ is based on the original study^[2]^; however this paper clearly shows that methylprednisolone actually had worse results than hyaluronic acid at 26 weeks. WOMAC scores are not mentioned in the original studies^[4,5]^ and score conversion has not been mentioned in the methodology section of the review article.^[1]^ Moreover, these original studies^[4,5]^ evaluate the efficacy of methylprednisolone before exercise and in addition to exercise for pain relief and this aspect is not mentioned by the authors in their article.^[1]^

In light of the above concerns about the inaccuracies in review methodology and conclusions, the *Medicine* editors are retracting this article.
